# Decoding a Substantial Set of Samples in Parallel by Massive Sequencing

**DOI:** 10.1371/journal.pone.0017785

**Published:** 2011-03-09

**Authors:** Mårten Neiman, Sverker Lundin, Peter Savolainen, Afshin Ahmadian

**Affiliations:** Science for Life Laboratory, Department of Gene Technology, School of Biotechnology, Royal Institute of Technology (KTH), Solna, Sweden; Technical University of Denmark, Denmark

## Abstract

There has been a dramatic increase of throughput of sequenced bases in the last years but sequencing a multitude of samples in parallel has not yet developed equally. Here we present a novel strategy where the combination of two tags is used to link sequencing reads back to their origins from a pool of samples. By incorporating the tags in two steps sample-handling complexity is lowered by nearly 100 times compared to conventional indexing protocols. In addition, the method described here enables accurate identification and typing of thousands of samples in parallel. In this study the system was designed to test 4992 samples using only 122 tags. To prove the concept of the two-tagging method, the highly polymorphic 2^nd^ exon of DLA-DRB1 in dogs and wolves was sequenced using the 454 GS FLX Titanium Chemistry. By requiring a minimum sequence depth of 20 reads per sample, 94% of the successfully amplified samples were genotyped. In addition, the method allowed digital detection of chimeric fragments. These results demonstrate that it is possible to sequence thousands of samples in parallel without complex pooling patterns or primer combinations. Furthermore, the method is highly scalable as only a limited number of additional tags leads to substantial increase of the sample size.

## Introduction

New generations of massively parallel sequencers have revolutionized the field of sequencing. With the dramatic reduction of sequencing cost and the ongoing development of new high throughput sequencers, the possibility of investigating hundreds of gigabases in a single sequencing run has come into effect. The throughput of recently developed sequencers, the HiSeq 2000 (Illumina) and SOLiD 4hq system (Life Technologies), makes it possible to, for instance, RNA-profile a couple of hundred samples in parallel while producing 10 million reads per sample. Targeted enriched cDNA libraries have also been shown to be an effective method for expression profiling [1], [2], [3] where massive sequencing could be used as readout with the possibility to screen thousands of samples in parallel. In addition, targeted highly multiplex amplification methods, such as molecular inversion probes [4], [5], GoldenGate [6] and trinucleotide threading [7], combined with massive sequencing is a powerful way of genotyping large sets of markers and samples at the same time [8]. In population genetics these platforms make it possible to tag and in parallel sequence highly polymorphic loci such as the MHC gene complex [9], [10], [11]. Retrieving genotype data from large cohorts is essential for studies aiming to investigate a chain of occurrences by studying stochastic events, such as mutations. The continuously growing sequencing throughput enables researchers to analyze more and more samples within the same experiment, hence creating a need for scalable and manageable protocols for preparing and typing these large sets of samples.

In order to sequence a huge number of samples in a single sequencing run, a stable and reliable method for identifying the generated reads is required. The introduction of DNA barcodes by PCR or by ligation has previously been described as methods to distinguish the origin of each read in a set of mixed sequences [12], [13], [14], [15], [16]. In this study we demonstrate how the sample pooling and identification procedures can become more reliable and less complicated, enabling larger experimental designs. In addition to the complexity of designing and performing highly multiplex experiments, the cost of reagents and the time of protocol executions often limit the experiments. By setting up an automated protocol utilizing third party reagents, we describe how both time and cost for sample processing prior to sequencing can be substantially reduced. We describe a pooling strategy able to produce an even spread of reads across the barcoded samples, addressing the growing need of multiplex genotyping.

The technique described in this work utilizes a combination of two tags to identify the origin of a certain read and thereby reduces the number for unique tags needed for a given number of samples. Instead of incorporating one identification tag at the end of the amplicon, a first tag is incorporated at both ends of PCR fragments and then a second tag is ligated to a pool of 96 samples originating from a 96-well PCR plate. By using this system the complexity of sample handling is greatly reduced and the need for making unique primer combinations for each sample is eliminated.

The method was tested on 52 indexed plates, allowing analysis of 4992 samples, by sequencing the 270 bp 2^nd^ exon of the highly polymorphic DLA-DRB1 gene using the 454 GS FLX Titanium Chemistry [17]. By taking advantage of this two tagging system we demonstrate that it is possible to readily sequence very large cohorts in parallel.

## Materials and Methods

### Samples and DNA preparation

The sample set consisted of a total of 4708 dogs and wolves: Animal Ethics Committee, The State Provincial Office of Southern Finland, ESLH-2009-07827/Ym-23; Federal Fish and Wildlife Permit Number MA053639-1; Endangered/Threatened Species Wildlife and CITES Appendix II Permit No. 09US222450/9; University of New South Wales *Care and Ethics Committee (ACEC)* Project 05/74A. 2059 of the samples were collected by FTA-cards, 819 were hair samples and the remaining 1830 were blood samples. The DNA from hair and blood was extracted as described by Savolainen *et al.* [18] while the FTA-cards (Whatman, Maidstone, UK) were prepared according to the manufacturer's instructions (using a 2 mm punch) with the exception that the washing volumes were adjusted to 100 µl. A Magnatrix™ 1200 (NorDiag ASA, Oslo, Norway) liquid handling robot was used for washing 96 pellets in parallel using sample specific tips and washing solution containers. After the final wash, the pellets were dried at 56°C for 10 minutes.

### Primers

DLA-DRB1 exon 2 primer sequencess were obtained from Kennedy *et al.* [19]. To distinguish the different samples in each plate, 96 pairs of DLA-DRB1 exon 2 primers with different tags were designed (supplementary table S1). The tagging sequences were obtained from Meyer *et al.* [13]. Tags were chosen to not contain any homo polymeric combinations and to differ from each other in at least three positions. 8 bases of identification sequence were used for the position-tags. The primers were synthesized by Thermo Scientific (Wilmington, DE, USA).

### Tagging by PCR

Touch-down PCR was performed in a total volume of 50 µl containing 1 U Platinum Taq DNA polymerase (Invitrogen, Carlsbad, Ca, USA), 1× Taq Platinum DNA polymerase buffer (Invitrogen), 1.5 mM MgCl_2_, 0.2 mM dNTPs and 0.2 µM of the forward and reverse primers, respectively. After initial activation at 95°C for 2 min the reaction was cycled through 14 cycles of touch-down starting at 95°C for 30 seconds, 62°C for 1 min and 72°C for 1 min., and lowering of annealing temperature by 0.5°C for each cycle making the last cycle anneal at 55°C. Then, depending on template source, 40 or 45 cycles of 95°C for 30 seconds, 55°C for 1 min and 72°C for 1 min followed and the reaction ended in a final extension of 10 min at 72°C. For FTA-card samples the number of post touch-down cycles were 45 and for blood and hair samples it was 40. About half of the PCR-products were examined by gel electrophoresis or capillary electrophoresis using the MCE®-202 MultiNA Microchip Electrophoresis System (Shimadzu Biotech, Kyoto, Japan).

### Pooling and clean-up

For each plate, 5 µl of each sample was pooled and 250 µl of the resulting mixture was applied to a QIAquick™ PCR clean up column (Qiagen, Hilden, Germany) and treated according to the manufacturer's procedures for PCR clean up and eluted in 30 µl of Qiagen elution buffer (EB). The concentrations of the cleaned pools were measured using Invitrogen's Quant-iT™ dsDNA BR kit. The concentration of each pool was then adjusted to 1 µg DNA/34 µl EB.

### Automated MID ligation and sequencing

52 pools were indexed using the extended set of 454 Multiplex Identifier (MID) adaptors for the GS FLX Titanium Chemistry (supplementary table S2). Adaptors were synthesized by Thermo Scientific. All steps of the library preparation were automated and performed using a Magnatrix™ 1200 Biomagnetic Workstation (NorDiag) capable of running custom made scripts as described previously [20] with the exception that the library reagents including end repair, ligation and fill in reagents were purchased from New England Biolabs (NEBNext End Repair Module, NEBNext Quick Ligation Module and Fill-in and ssDNA Isolation Modules) (New England Biolabs, Ipswich, MA, USA). The quality of each library was examined after ligation using Experion 1k DNA analysis kit (BioRad, Herculees, CA, USA). Three equimolar pools were made from the 52 adaptor indexed libraries and single stranded DNA was isolated from each pool. The emulsion PCR titration by quantification, amplification and sequencing were performed using the 454 GS FLX Titanium Chemistry according to the manufacturer's instructions. Each library pool was sequenced on a separate lane using a total of three lanes in a four-lane setup.

### Data analysis

The generated reads were sorted by their plate- and position-tags into individual folders and the reading direction were normalized using an in-house developed BioPerl [21] script. All reads corresponding to each of the individuals were separately aligned using MUSCLE [22] generating one alignment per individual. Starting from these sequence alignments, one or two consensus sequences were generated for each individual depending on if the individual was homozygous or heterozygous respectively. To compensate for pyrosequencing errors in homo polymeric regions, the generated consensus sequences were aligned against a reference sequence and deletions in the sequencing data were corrected.

## Results and Discussion

The introduction of massively parallel sequencing platforms has opened up entirely new possibilities in all fields of functional genomics. However, the capacity of these platforms greatly exceeds the needs in most studies involving targeted profiling and typing. In an attempt to better exploit the capacity of these systems and reduce the cost per sequenced sample, indexing procedures has been introduced, allowing multiplex identification and sorting of over 100 samples [23], [24]. However, increasing the sample size to thousands necessitates additional tagged adaptors leading to preparation of thousands of libraries, which is time consuming and expensive. This problem has been addressed by Erlich *et al.* [14] by implementing a sample-pooling pattern combined with tagging of the pools and Galan *et al.* [16] by combination of tags in the forward and reverse PCR primers. Although these studies demonstrated that the number of unique tags does not necessarily need to match the number of samples, they considerably increase the complexity of sample handling by employing experimentally complicated sample or primer mixing procedures. In this study we present a two tagging strategy that employs a combination of two tags, added in two steps, which not only enables accurate multiplex analysis of thousands of samples in parallel, using a reduced number of tags, but also reduces the complexity of sample handling.

**Figure 1:**
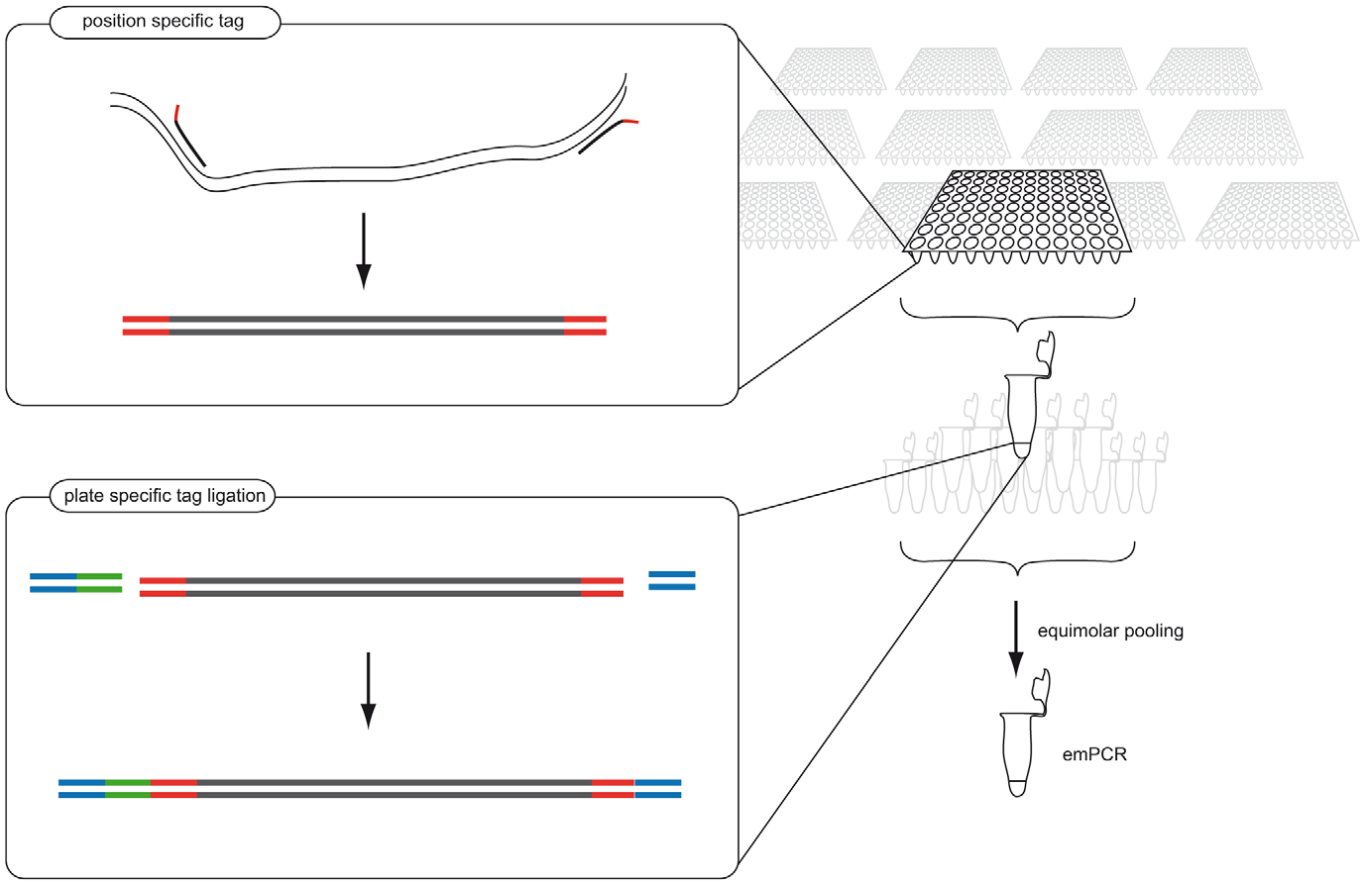
The tagging procedure. 96 tagged primer pairs are designated to each position in a 96-well PCR plate grid giving one specific tag for each position, referred to as position tags (shown as red). Sets of 96 samples are tagged and amplified hence, giving N/96 plates (where N is the total number of samples). All products within each plate are pooled and a second tag, denoted plate tag (shown as green) together with the sequencing adaptors (shown as blue), is ligated to the pool. The plate-indexed products are then pooled and amplified prior to sequencing.

The principle of the two tagging strategy is depicted in figure 1. The tags are added in two steps. First, the target is amplified by tagged PCR primers giving amplicons that are tagged in both ends (denoted position-tags). By using 96 position-tags and strictly handle them in a 96-well PCR-plate, 96 samples are uniquely tagged in a single round. The PCR products from a plate are then pooled and a second tag (denoted plate-tag), which is incorporated next to the sequencing primer, is ligated to the pooled samples. The degree of sample multiplexity is thus substantially increased by adding up indexed plates. The number of required tags can be described as *tags = (N/96)+96*, where N is the total number of samples (so that N/96 expresses the number of required plate-tags while the constant 96 is the number of position tags in the PCR plate). However, if more than one sequencing lane is utilized, the number of tags is reduced to tags = (N/L*96)+96 where L is the number of lanes used for sequencing the sample set. In this study, N was set to 4992 and L to 2, giving a total of 122 (26+96) tags.

To prove the concept of the two tagging approach, the 2^nd^ exon of the DLA-DRB1 gene in dogs and wolves was amplified using the 96 position-tags in 52 plates. Out of the 4992 wells, samples were added to 4708, while 284 wells were used for negative PCR controls. After gel electrophoresis, the number of successful PCR products was estimated to 3700 (a success rate of about 79%). The success rate of the PCR was largely dependent on the quality of the samples. For example, 59% of the DNA samples originated from hair and 85% from FTA cards resulted in detectable products. The rate of successfully amplified samples would however increase if the quality of all samples were checked prior to amplification. In addition, two of the position-tagged primers (D11 and D12 (supplementary table S1)) did not give any products at all and position-tagged primers C3 and G9 performed poorly. This is probably due to poor primer synthesis and since the position-tags exist in all 52 plates, over 150 of the failed PCR reactions could be related to these primers.

**Figure 2:**
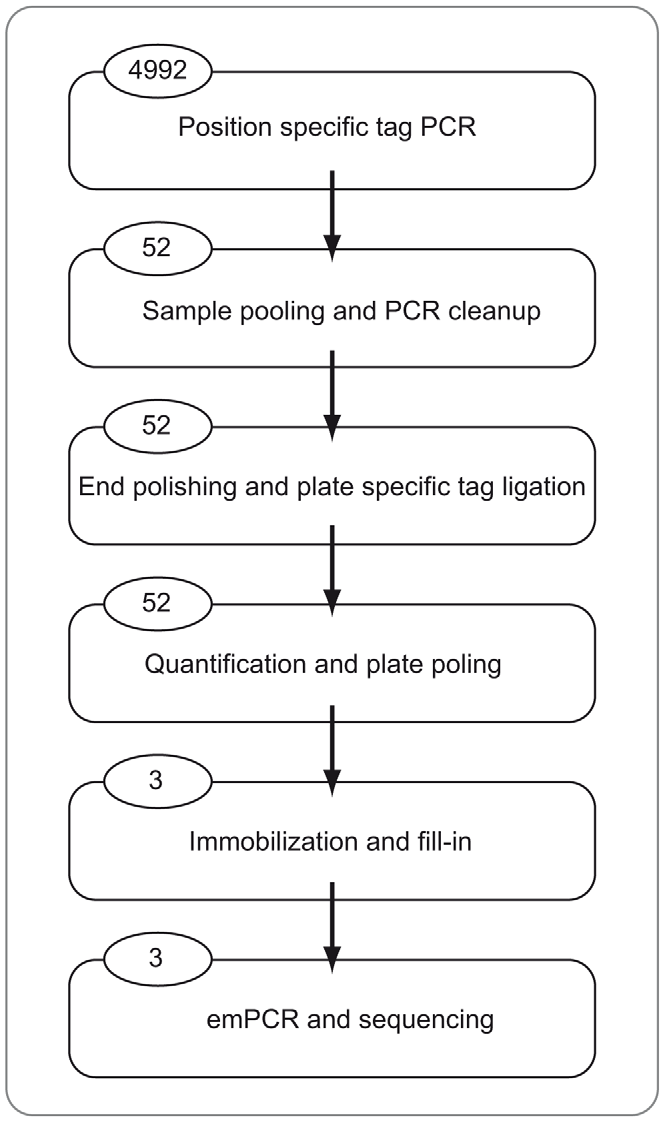
The workflow. Figures in circles indicate the number of samples that are handled within each step showing the reduction of sample handling complexity. The first step is conducted plate wise, marking 96 samples at a time with a position (specific) tag. In the second step pooling of the 96 samples in each plate is performed and the pooled samples are subjected to spin column PCR cleanup. This step is followed by the third fully automated step of end polishing, phosphorylation and plate (specific) tag ligation. The concentration of the ligation products are measured and the plate pools are equimolarly pooled into one sequencing library for each lane. The immobilization of sequencing libraries onto paramagnetic beads and fill-in reaction is fully automated. The workflow ends with standard emPCR and sequencing.

**Figure 3:**
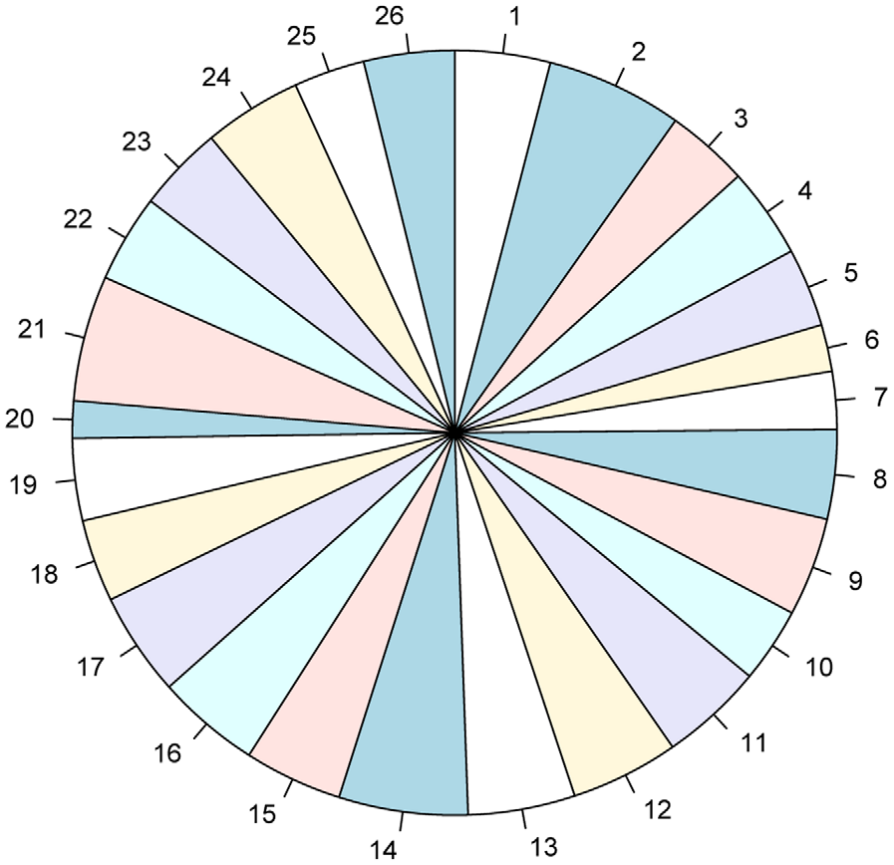
Distribution of reads across the MIDs. Pie chart showing an even read distribution of the plate specific tag, illustrating successful equimolar pooling of ligated fragments prior to immobilization, fill-in and emPCR. Since the experiment was designed for two lanes, each plate tag was used for tagging two plates and hence, each sector represents 2*96 = 192 samples.

After initial PCR amplification, the library pooling strategy was deployed, where each library was pooled prior to ssDNA isolation, enabling time and cost effective sample processing (figure 2). Worth mentioning, to save time and reagents, no concentration measurement of the 4992 PCR products was done. The 52 libraries could be processed in two days using an automated setup, and using third party reagents substantially reduced the cost. Uneven read distribution between indexed libraries is a common problem with increasing degree of multiplexing, raising high requirements on concentration determination. By using an automated electrophoresis station it was possible to distinguish the ligated amplicon peak from the non-ligated peak (supplementary figure S1), allowing for correct quantification of ligated amplicons and volume adjustment and thereby enabling accurate library pooling. With this set-up an even read distribution was obtained (figure 3).

The 52 libraries were sequenced on the 454 GS FLX Titanium Chemistry using three out of four lanes, generating a total of 700,000 reads. As mentioned above, the project was designed to employ 2 lanes (L = 2) but as we managed to access three lanes, the libraries were divided into three emPCR and sequencing lanes instead of two (see figure 2 and supplementary table S2). For 285,000 reads, the read-lengths were long enough to identify the plate-tag and both position-tags. The low rate of useable sequence reads could be explained by the use of a strict criterion not allowing sequence errors in the tags and the fact that the target region harbors a high number of homopolymeric sequences (a well known problem with Pyrosequencing chemistry). 10% of these sequence-reads showed different position-tags in the ends, indicating chimeric formation. These chimers are most probably formed from different single-stranded PCR-products (from different samples) that are recombined when amplicons within each plate are pooled and extended to completion during the end-polish reaction. To minimize this effect PCRs with fewer cycles could be performed (reducing the amount of single stranded amplicons) or the end-polish reaction could be skipped (by employing a polymerase that lacks terminal transferase activity in the PCR). However, the fact that the inner position-tags are incorporated in both ends of the sequencing templates makes it possible to detect chimeric sequences from different samples and exclude them from the data analysis. This demonstrates that our straightforward approach to detect chimeric sequences is needed for obtaining correct results during multiplex sequencing and sorting of polymorphic genes. These erroneous constructs would have gone undetected or at least difficult to detect employing previously mentioned tagging methods [16]. To further investigate the extent of multi-sample chimeric sequence formations, a library was created in which the plate-tags were incorporated by a second PCR (using the C and D handles, see primer sequences in supplementary table S1) after sample pooling (instead of ligation). This means that there were 96 samples in each plate-tagging PCR and thus the extent of chimeric products was expected to increase. After sequencing, chimeric sequences were observed in 97% of the reads where all tags could be identified (data not shown). We therefore strongly recommend that samples should not be pooled before a PCR reaction when sample sorting and individual genotyping is the aim of the study. Allelotyping of pooled samples [8] is not included in this recommendation since the aim of these studies is to estimate and compare allele frequencies in different cohorts.

**Figure 4:**
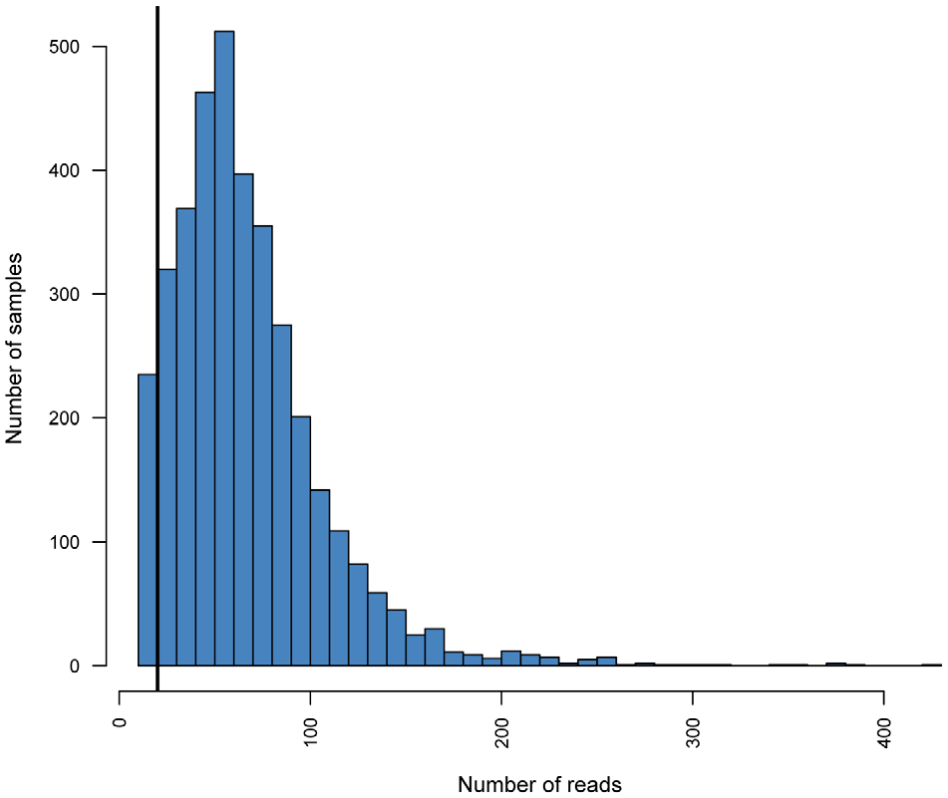
Distribution of reads across the samples. Histogram showing the distribution of reads per sample for the 3700 successfully amplified samples. The vertical line indicates the breakpoint of 20 reads required for accurate genotyping.

**Figure 5:**
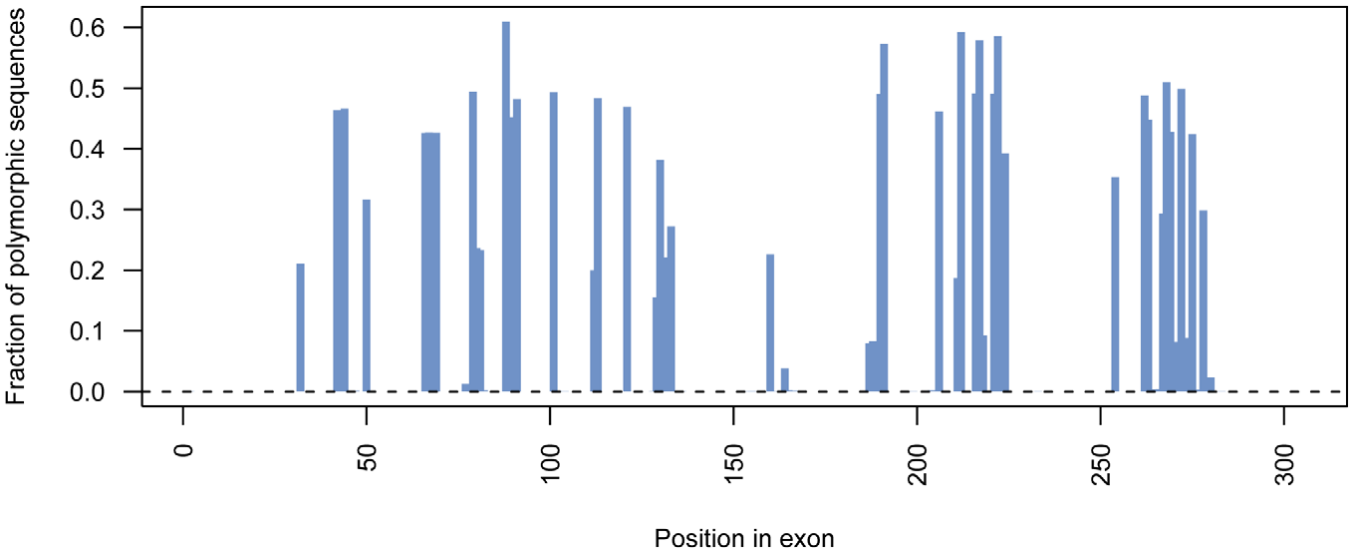
Polymorphisms. Indicating the polymorphic positions across the target exon. All generated consensus sequences have been aligned together and the diversity of each position in the alignment has been calculated. The bars indicate the fraction of the aligned consensus sequences that differs from the most common allele at a certain position.

A ten-fold difference between the most and the least sequenced position-tags was observed (data not shown). However, since each position-tag is incorporated in 52 samples, there should be an even spread of sample quality across the position-tags, hence less difference would be expected. The difference in sequence depth within the position-tags is therefore more likely an effect of primer qualities in combination with the fact that different tag sequences could affect the primer annealing by forming secondary structures. Still, the majority of the PCR products were correctly genotyped. The minimum requirement for correct genotyping was set to 10 reads per allele resulting in 20 reads per individual [25]. Out of the estimated 3700 PCR-products, 3465 generated more than 20 reads (figure 4). To investigate how the polymorphisms are distributed across the sequenced exon, all generated consensus sequences were aligned and the fraction of polymorphisms for each position was plotted (figure 5). This resulted in a number of hot spot positions where the polymorphisms are concentrated.

To conclude, we here demonstrate a simple, robust and reliable method for sequencing thousands of samples in parallel in one single sequencing run. The method is robust enough to omit the time and reagent consuming step of equimolar pooling at the individual level by performing equimolar pooling of 96 samples at a time after introduction of the second plate specific tag. The need for making unique primer combinations for each sample is replaced by having the same 96 position specific primer pairs for all PCR plates. By strictly handling the PCR tagging procedure in 96-wells format and by automating the ligation of plate-tags and library preparation, it is possible to increase the number of samples without affecting the sample handling complexity. This higher control over pipetting errors combined with automated library preparation is one of the main strengths of the presented method since this approach does not require less number of unique primers compared to the competing methods. We have shown that it is possible to obtain sufficient sequence depth for 94% of the successfully amplified samples when running at a multiplexing level of 4992 tag combinations on the 454 sequencing system. Furthermore, incorporation of the same position-tags at both ends of the fragments allows detecting chimeric sequences from different samples that is a prerequisite for accurate identification of samples. We believe that the reliability of the method combined with scalability makes it suitable for sequencing targeted enriched DNA or RNA of even greater sample sizes on platforms such as HiSeq 2000 and SOLiD where the number of reads per run is in the magnitude of 2000 times greater than the 454 system.

## Supporting Information

Figure S1 **Electropherogram of ligated product.** Indexed library concentration determination. A characteristic library electropherogram illustrating the main ligated product peak that was used for quantification, separated from the non-ligated smaller peak not taken into account when the concentration was determined.
(TIF)

Table S1 **Indexed PCR primers.** The 96 primer pairs used for position specific tagging by PCR. The gene specific regions are situated in the 3′-ends of the primes, the tag sequence in the middle and at the 5′-end universal handles are included.
(XLS)

Table S2 **Concentration of ligated products.** The 52 pooled PCR plates were indexed using 26 MID adaptors. Due to poor performance, related to primer synthesis, some MIDs were excluded hence, the lack of some numbers in the MID column. Three library pools (1, 2 and 3) were made, each sequenced on a separate lane. Concentrations were determined for the large (ligated) peak.
(XLS)
